# Vehicle Occupant Detection Based on MM-Wave Radar

**DOI:** 10.3390/s24113334

**Published:** 2024-05-23

**Authors:** Wei Li, Wenxu Wang, Hongzhi Wang

**Affiliations:** Collage of Information, North China University of Technology, Beijing 100144, China; wangwenxu@mail.ncut.edu.cn (W.W.); 18310003825@163.com (H.W.)

**Keywords:** millimeter-wave radar, frequency-modulated continuous-wave technology, vehicle occupant detection, range–azimuth heatmap, convolutional neural networks

## Abstract

With the continuous development of automotive intelligence, vehicle occupant detection technology has received increasing attention. Despite various types of research in this field, a simple, reliable, and highly private detection method is lacking. This paper proposes a method for vehicle occupant detection using millimeter-wave radar. Specifically, the paper outlines the system design for vehicle occupant detection using millimeter-wave radar. By collecting the raw signals of FMCW radar and applying Range-FFT and DoA estimation algorithms, a range–azimuth heatmap was generated, visually depicting the current status of people inside the vehicle. Furthermore, utilizing the collected range–azimuth heatmap of passengers, this paper integrates the Faster R-CNN deep learning networks with radar signal processing to identify passenger information. Finally, to test the performance of the detection method proposed in this article, an experimental verification was conducted in a car and the results were compared with those of traditional machine learning algorithms. The findings indicated that the method employed in this experiment achieves higher accuracy, reaching approximately 99%.

## 1. Introduction

Vehicle occupant detection technology is used in a wide range of applications. In the area of vehicle active safety, it can help vehicles achieve automatic emergency braking, collision warning, and other functions. When passengers are detected in the car, the car system will automatically adjust the braking distance and force to avoid or reduce injuries caused by crashes. In the area of vehicle passive safety, vehicle occupant detection technology can assist the vehicle system in adjusting the force and angle of airbags to better protect passengers’ heads and chests. Furthermore, this technology can also be used to realize the functions of automatic seat adjustment and automatic air conditioning temperature adjustment, improving the comfort of passengers. With the continuous development of autonomous driving technology, it will play a more important role in the field of automotive safety [1]. By working with other vehicle systems, it can more accurately estimate the status and needs of passengers, thereby achieving a more intelligent and personalized driving experience.

Currently, there are two main ways of conducting vehicle occupant detection: contact and non-contact. Contact methods for detecting vehicle occupants mainly involve installing sensors on the seats to detect whether the seats are occupied. For example, pressure sensors can be installed in a logical position on the seat [2,3,4,5,6]. Pressure sensors mainly convert pressure signals into voltage signals that carry pressure information according to certain conversion rules, e.g., detecting the passenger weight, pressure, and acceleration information, and then inferring whether there is a stranded target in the vehicle based on logic. When it is determined that there are stranded people in the vehicle, a warning will be sent through communication between the vehicle and the mobile phone for a timely reminder to avoid the tragedy. However, there are some loopholes in this method, such as the pressure sensors not being suitable for installation on vehicle seats or not being able to distinguish the difference between a passenger and a heavy object placed on the seat, among other things. A method based on electric field or capacitance induction [7,8,9,10] uses the electrical shielding effect to solve the above problems [11], but it is more sensitive to vehicle vibration and easily causes false alarms.

Non-contact methods for detecting occupants in vehicles mainly utilize optical systems and electromagnetic waves to determine whether there are any passengers left inside the vehicle. The occupant detection method based on optical sensors that is used to detect the number of passengers, which also involves installing an artificial intelligence camera in the car [12,13,14], is a feasible solution. There is already advanced technology designed to achieve target detection with cameras. However, camera sensors rely heavily on light and are unable to perform well when there is a significant change in light levels. Additionally, with the increasing emphasis on personal privacy, this scheme is not suitable for promotion in such a private space inside a car. One study [15] proposes a method that combines deep learning and thermal imaging. Thermal imaging pictures are used as the input for the convolutional neural network to obtain a high-precision passenger counting result. However, the cost of using this sensor is high, and it is very sensitive to changes in vehicle temperature. The test results exhibit some inaccuracies between the detection results and the experimental conclusions obtained in an ideal environment, and they may not be able to detect passengers in the car when the internal temperature is high.

Compared to the above methods, using radar systems as sensors for occupant detection in vehicles has the following advantages:

1. Unaffected by light: Radar systems utilize electromagnetic waves for detection, allowing them to operate normally regardless of light intensity, even in environments with no light. However, cameras rely on light, and their detection effectiveness may be impacted in conditions of insufficient or excessive light.
2. Strong penetrability: Radar systems can penetrate through some non-metallic materials, such as clothing and blankets, enabling the detection of occluded occupants.
3. Privacy protection: Radar systems do not require capturing the image information of occupants during detection, thus better protecting the privacy of vehicle occupants.
4. Strong tamper resistance ability: Radar systems have strong anti-interference abilities and can resist external factors such as electromagnetic interference and noise interference. Other sensors may be affected by these interferences, causing a decrease in detection effectiveness.

In short, radar systems have the advantages of high integration, low cost, and low power consumption, and they are not affected by weather conditions and do not infringe on personal privacy [16]. Therefore, using radar as a sensor can provide reliable and accurate detection for passengers in vehicle environments.

Currently, most methods for detecting people in vehicles based on radar sensors utilize radar systems for vital sign detection [17,18,19]. However, the shaking of the human body, the vibration of vehicles, and the movement of inanimate objects can all generate Doppler signals, which may conceal or misreport them as vital signs of human respiration and heartbeat. Therefore, it is challenging to determine whether there are any passengers in a vehicle solely by using radar sensors to detect vital signs. One study [20] proposes a low-cost pulse millimeter-wave radar-based system for occupant detection in vehicles, but due to its low transmission power, it is necessary to install radar sensors in front of each seat to ensure detection accuracy. Another study [21] employs a 2.45 GHz continuous-wave radar to collect Doppler spectra from passengers, extracting features from human Doppler signals using machine learning methods for occupant detection in vehicles. Nevertheless, extracting Doppler information solely from passengers on the seat cannot provide their location and number. Additional studies [21,22] utilize millimeter-wave radar to obtain the range, speed, and azimuth information on the passengers; generate radar point cloud maps of passengers; and utilize these point cloud maps for vehicle occupant detection. However, when the above method is used for occupancy detection in vehicles, the discrete distribution of point cloud data increases the complexity of the data and the difficulty of processing. Additionally, lower point cloud density and resolution can make it difficult to distinguish individual targets. To intuitively distinguish between different targets, not only is a high-resolution radar required, but a very complex signal processing method is also needed.

Compared to point cloud maps, heatmaps have lower data complexity and processing difficulty, and they are more intuitive in displaying target information. Given the lack of current radar sensor-based vehicle occupant detection methods, this paper uses millimeter-wave radar to detect vehicle occupants by obtaining radar heatmaps and combining them with neural networks. We process the Echo signal that is collected by millimeter-wave radar through Range-FFT and DoA estimation algorithm to obtain information, such as the distance and angle of the target relative to the radar, thus further generating range–azimuth heatmaps to visually display the current status of personnel inside the vehicle. Finally, by utilizing the method that combines radar signal processing technology with deep learning algorithms, we input the range–azimuth heatmaps of passengers in different seating states into the Faster R-CNN neural network to extract feature information, as well as to predict the number of occupants and their locations.

The contributions of this paper are as follows:

- The proposal of an in-vehicle occupant detection method based on millimeter-wave radar, which improves privacy and reliability;
- The collection of radar heatmaps for target feature extraction, reducing the complexity of data processing;
- The utilization of neural networks to judge passenger information, providing a more intuitive representation of accuracy;
- The use of experimental verification in real in-vehicle environments to ensure the rigor of the experiments.

The remainder of this paper is organized as follows. The second section introduces the theory of occupant imaging in vehicles based on FMCW radar. It provides a method for extracting target range and azimuth information from MIMO FMCW radar and shows the range–azimuth heatmap. The third section presents the occupant detection model based on the Faster R-CNN neural network. It discusses the network structure of Faster R-CNN and provides the model design for this experiment. Finally, the fourth section provides the conclusions and a discussion of future work.

## 2. System Design and Principle

This section describes the occupant detection process in a vehicle based on millimeter-wave radar, provides the basic information of MIMO FMCW radar, and the theories of extracting target range and azimuth information. It also expounds the process of obtaining range–azimuth heatmaps of rear passengers.

### 2.1. System Composition

**Figure 1:**
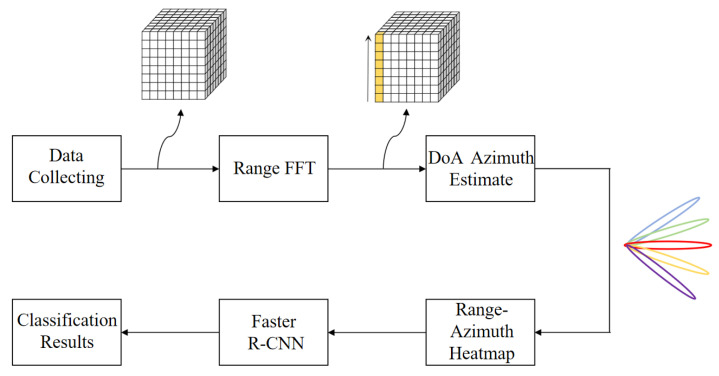
System construction.

The vehicle occupant detection system construction based on millimeter-wave radar proposed in this paper is shown in Figure 1.

The radar cube data of the target are collected using the radar module, distance FFT and DoA angle estimation are performed on the collected data, information such as the distance and angle of the target relative to the radar is obtained, a range–azimuth heatmap is generated, and the heatmap is sent to the neural network for training to obtain the training results.

### 2.2. FMCW Radar

**Figure 2:**
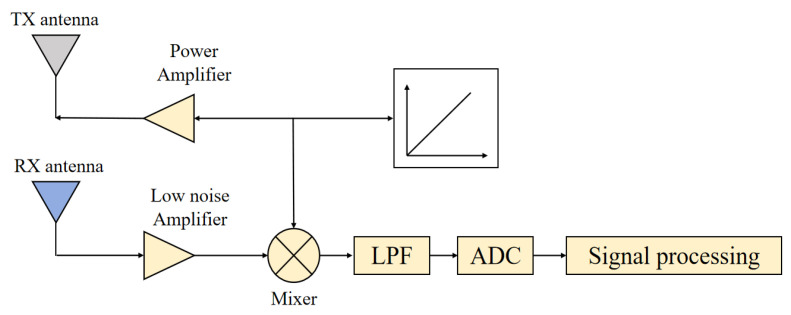
The working principle of radar.

The millimeter-wave radar system used in this paper operates using frequency-modulated continuous-wave technology, the system consists of a transmitting antenna, a receiving antenna, a filter, a mixer, a waveform generator, signal processing modules, and other components. When the radar is working, the waveform generator generates a linear frequency modulation signal, part of which is amplified by the power amplifier to generate a transmit signal, and the other part is mixed with the receive signal in the mixer to generate an intermediate frequency signal, which is then sampled by an AD converter to obtain a digital intermediate frequency signal. After signal processing, basic target information, such as distance and angle, can be obtained. The schematic diagram is shown in Figure 2.

**Figure 3:**
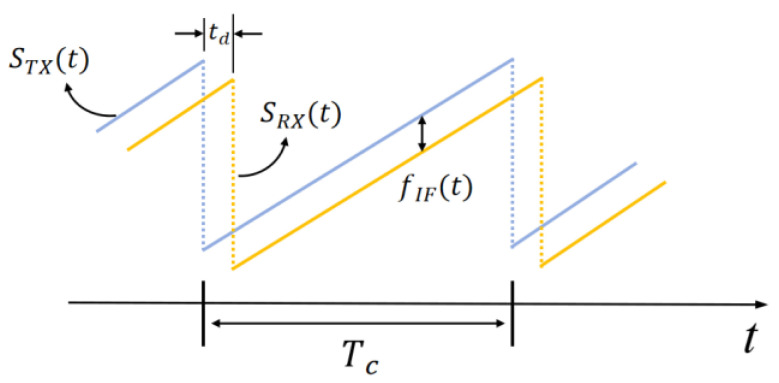
Relationship between the transmitting signal and the receiving signal.

The transmitting and receiving signals of radar are both sawtooth waves, and the receiving signal has a fixed time delay dt. The relationship between the transmitting signal and the receiving signal is shown in Figure 3.

The transmitting signal in a single transmit cycle can be expressed as the following:

(1) $$S_{TX}\left(t\right)=A_{TX}cos\left(2\pi f_{c}t+\pi kt^{2}\right)$$

Among them, $\;k=B/T_{c\;}$ is the chirp rate, $\;T_{c}$ is the signal period,  B  is the signal bandwidth, $f_{c}$ is the carrier frequency, and $A_{TX}$ is the amplitude of the transmitting signal.

Assuming that the distance of the target from the radar is  R, the delay from transmitting signals to receiving antennas is $t_{d}=\frac{2R}{c},$ and so the echo signal of the target received by the radar can be expressed as the following:

(2) $$S_{RX}\left(t\right)=A_{RX}cos\left(2\pi f_{c}\left(t-t_{d}\right)+\pi k{\left(t-t_{d}\right)}^{2}\right)$$

After inputting both transmitting signal $S_{TX}\left(t\right)$ and echo signal $S_{RX}\left(t\right)$ into the mixer at the same time, and after filtering the high-frequency signal with a low-pass filter to obtain the IF signal $S_{IF}\left(t\right)$, the following can be expressed:

(3) $$S_{IF}\left(t\right)=\frac{1}{2}A_{TX}A_{RX}cos\left(2\pi k\left(t_{d}t-\frac{1}{2}t_{d}^{2}\right)+2\pi f_{c}t_{d}\right)$$

**Figure 4:**
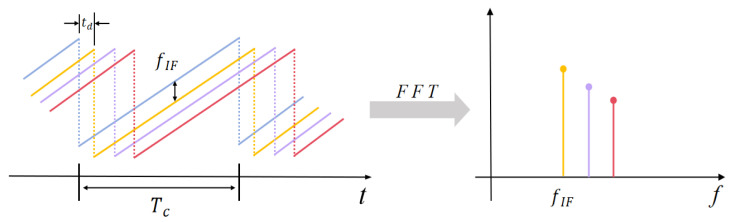
Fourier transform on IF signals.

Due to the very small value of $t_{d}$ during the actual measurement process, meaning the value of $t_{d}^{2}$ is negligible, the frequency of the IF signal can be similarly expressed as the following:

(4) $$f_{IF}=kt_{d}=\frac{B}{T_{c}}\cdot \frac{2R}{c}$$

c is the speed of light. During the target detection process of FMCW radar, unable to directly obtain the time delay $t_{d}$, it uses the frequency of IF signals instead of time delay for distance estimation. So, when the radar detects multiple objects, performing Fourier transform on IF signals can separate different frequencies $f_{IF}$ and their corresponding amplitude sizes, as shown in Figure 4.

So, the distance between the target and the radar can be obtained as the following:

(5) $$R=\frac{c}{2B}\cdot f_{IF}\cdot T_{c}$$

**Figure 5:**
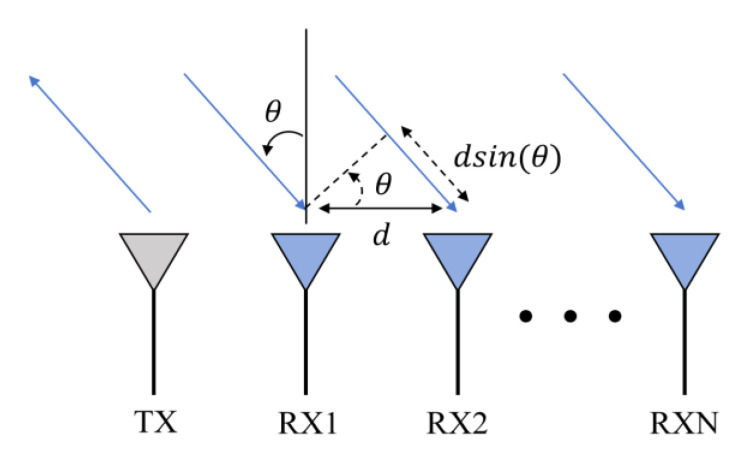
FMCW millimeter-wave radar angle estimation theory.

This paper uses the DoA algorithm to solve the FMCW radar angle estimation problem. DoA estimation is known as the arrival angle estimation, and it seeks to determine the direction of electromagnetic waves arriving at the antenna array. The basic idea of the DoA algorithm is to use the phase difference between the antenna arrays for angle estimation, one transmitting antenna that corresponds to at least two receiving antennas, the wave path difference between the target, and each receiving antenna. This will cause a change in the peak phase of the echo signal, using the phase difference, and the angle of the target relative to the radar can then be calculated. The angle estimation theory of FMCW millimeter-wave radar is shown in Figure 5.

For different return antennas, the phase difference is calculated as follows:

(6) $$∆\Phi =\frac{2\pi ∆d}{\lambda }\;\;\;\;\;\left|∆\Phi \right|<180^{\circ }$$

From this, ∆d=dsin(θ),  d  is the range between the two antennas,  θ  is the angle of arrival, and λ is the wavelength, so the calculation formula of the angle of arrival can be represented as the following:

(7) $$\theta ={sin}^{-1}(\frac{\lambda ∆\Phi }{2\pi d})\;$$

**Figure 6:**
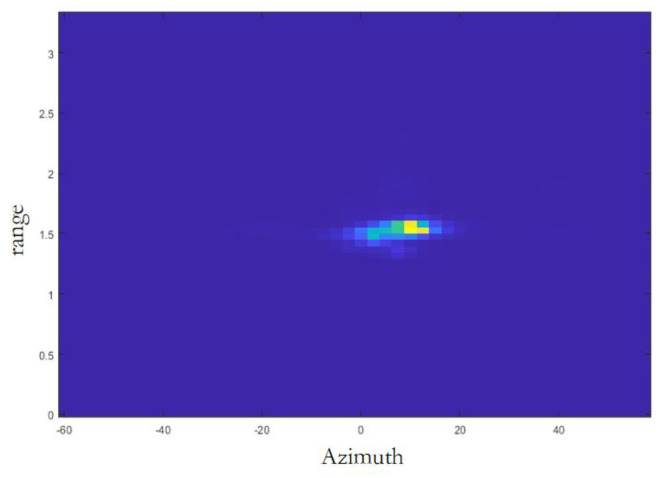
Range-azimuth heatmap.

The processing cell collects samples from the analog-to-digital converter (ADC) during each chirp rise time quantum. Before the samples are inputted to the Range-FFT module, in each RX channel, the data are reorganized into a two-dimensional array, and the data from the same chirp exist in one row of this two-dimensional array. Then, the Range-FFT module performs windowing and FFT operations. The output of Range-FFT will be inputted into the angle estimation module to calculate the angle of the target relative to the radar. After performing DoA spectral estimation to calculate the 2D heatmap of the frame, the MUSIC algorithm is used to calculate the covariance matrix of the received signal by averaging the received signal matrix over time. The covariance matrix is then decomposed into eigenvectors corresponding to the signal space and noise space directions, as well as eigenvalues corresponding to the energy magnitude in each direction. Based on the magnitude of the eigenvalues, the eigenvectors are divided into a signal subspace and a noise subspace. The signal subspace is composed of eigenvectors corresponding to the largest eigenvalues, while the noise subspace is composed of the remaining eigenvectors. A spatial spectral function is constructed using the noise subspace. Since the direction of the signal corresponds to the direction of arrival of the noise, the spatial spectral function will have a peak at the direction of arrival of the signal. By searching for the peak of this spatial spectral function, the direction of arrival of the signal can be estimated, thereby forming radar image information of the target. In clutter-rich environments, especially indoor environments, detecting objects with small RCS, such as pedestrian and life-form objects, can be buried under the strong interference from the clutters and become difficult to detect. So, before generating the range–azimuth heatmaps, clutter removal is performed. The DC component for each range bin is then estimated, across chirps in a frame, and the estimated DC component is subtracted for each range bin. The range–azimuth heatmap of one frame is shown in Figure 6. Finally, these range–azimuth heatmaps are inputted into the Faster R-CNN neural network to predict the number of passengers and their occupied seats.

## 3. Occupant Detection Model Based on Faster R-CNN

Faster R-CNN is a deep learning neural network model that proposes the concept of candidate region generation and integrates modules such as feature extraction, candidate region generation, classification, and regression into a convolutional neural network. Faster R-CNN is a two-stage algorithm which usually includes two stages: generating a set of candidate regions, and then classifying and accurately locating these candidate regions. Compared to traditional one-stage algorithms that directly generate bounding boxes and class probabilities on the image, two-stage methods often achieve higher levels of accuracy. At the same time, strategies for dealing with scale and position variations are often included in the candidate region generation stage, which can better handle small and overlapping targets in the image [23]. In addition, compared to classic algorithms in the field of object detection such as FPN, SPP-Net, Mask R-CNN, etc., Faster R-CNN has a simpler structure and can adapt to different application scenarios by modifying the parameters of RPN or using different feature extraction networks as feature extractors, which have good flexibility.

In this experiment, the input for the Faster R-CNN neural network is a millimeter-wave radar range–azimuth heatmap. Compared to the traditional point cloud map, the heatmap provides a more intuitive display of target information, representing different data values through color or brightness changes. This feature enhances the model’s robustness to changes in input data. Even if there is some disturbance in the input data, the model can still accurately identify and locate the target.

### 3.1. Network Structure

**Figure 7:**
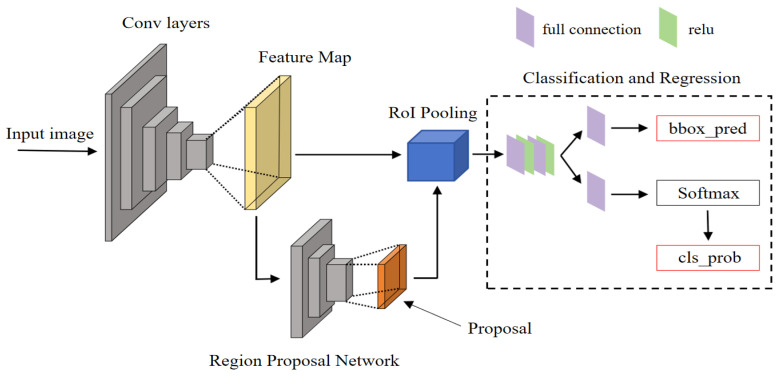
Faster R-CNN network structure.

The Faster R-CNN network framework consists of four parts: the feature extraction network, the RPN network, the RoI pooling layer, and the classification and regression module [24]. The basic network framework is shown in Figure 7.

The Faster R-CNN network first utilizes a feature extraction network to obtain the feature map of the input image, which is then divided into two parts: one part is passed forward as the input to the RoI pooling layer, while the other serves as the input to the RPN candidate region generation network. In the RPN network, the previously inputted feature map is used to generate candidate target regions, partitioning the input feature map into multiple regions of interest and generating a series of candidate regions through operations (such as classification and classification frame regression). Compared to traditional methods for generating candidate boxes, like Selective Search, it can more accurately predict the target position. Then, the output results of the first two parts are simultaneously fed into the RoI pooling layer, where candidate regions generated by the RPN network are collected and extracted from the feature maps of the first part. The output is sent to the fully connected layer to continue with classification and regression. In the classification and regression module, we use a Softmax classifier to achieve target recognition and classification, and we perform bounding box regression to confirm the final accurate position of the detection box [25].

### 3.2. Model Design

**Figure 8:**
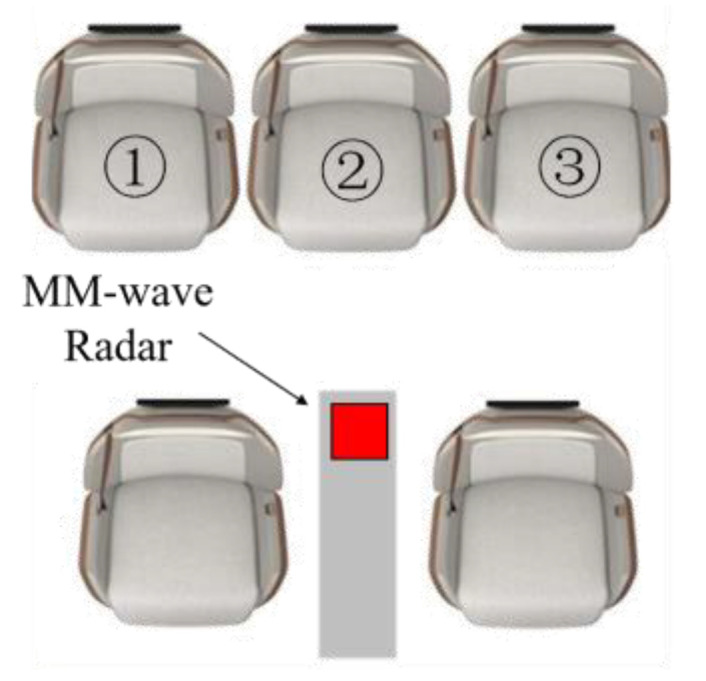
Radar placement position.

In this experiment, in order to fully cover the rear of the vehicle with the radar detection range and achieve the best testing effect, we will set the millimeter-wave radar at the position shown in Figure 8.

Here, 1, 2, and 3 represent the three seats in the back row from left to right. When a passenger is seated in one of the seats, the corresponding number of the seat is displayed as 1. If the seat is empty, the corresponding number of the seat is displayed as 0. For example, when there are passengers in the left and right positions of the back row and no passengers in the middle position, the current seating situation is 101. Table 1 shows the eight seating situations of the three seats in the back row in this experiment.

**Figure 9:**
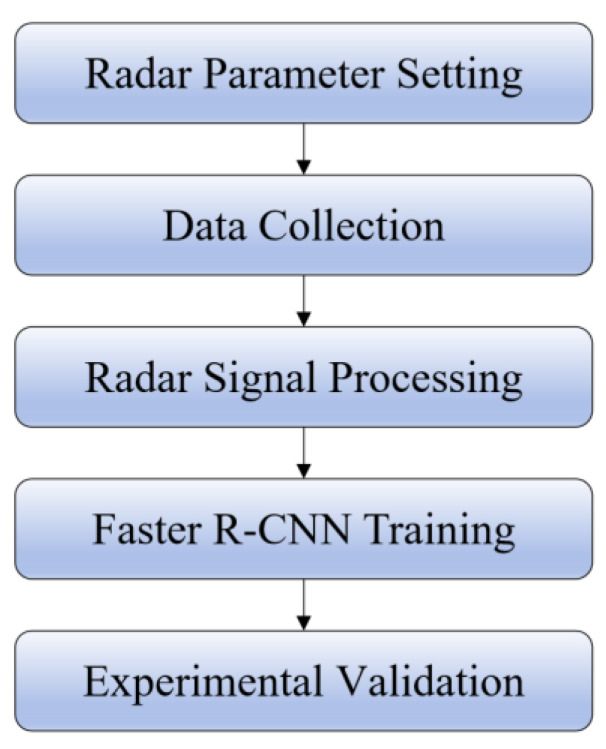
Experimental flow.

In this experiment, a total of nine people were invited for data collection. Groups of three were divided into three groups to enhance the data generalization. Then, 500 frames were collected per group for each situation, and a total of 12,000 frames were captured over 200 min. The experimental flow is shown in Figure 9.

**Figure 10:**
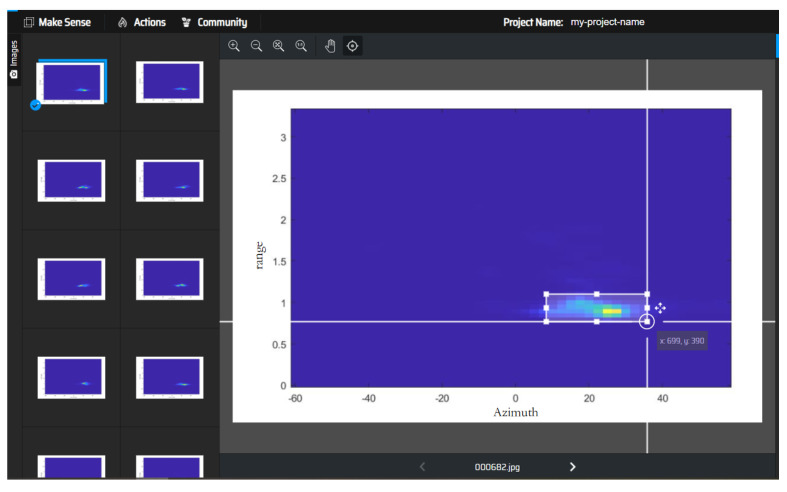
Make Sense interface.

Firstly, radar was used for data collection and a training set was established. The content of the dataset used range–azimuth heatmaps for different drop cases. The images were labelled in the training set. For this experiment, we chose the Make Sense image labeling tool to label each sample in the dataset. The labeling interface is shown in Figure 10.

The labeled range–azimuth heatmaps are placed into the training set for Faster R-CNN. Commonly used backbone feature extraction networks include VGG, ResNet, Xception, etc. This experiment uses the ResNet50 network as the backbone feature extraction network. The network will fix the size of the input image to 600 × 600. CUDA is then set to True, which means the GPU is used to accelerate calculations. The maximum learning rate is set as 0.0001, and the minimum learning rate is set as 0.01 of the maximum learning rate. For the setting of anchor_size, this experiment is set to [8, 16, 32], with each number corresponding to three prior boxes, resulting in nine prior boxes for each feature point. According to this setting, the generated prior box widths and heights are reported in the following: [90, 180], [180, 360], [360, 720], [128, 128], [256, 256], [512, 512], [180, 90], [360, 180], and [720, 360]. This paper uses the Adam optimizer to optimize the algorithm which combines the advantages of AdaGrad and RMSProp optimization algorithms. It is simple to implement, computationally efficient, has relatively low memory requirements, and the update of parameters is not affected by the scaling transformation of the gradient, making the optimization process more stable. Meanwhile, in this paper, we use the cosine annealing method, which is able to provide different learning rates at different stages of training to adapt to the needs of model training. At the beginning of training, higher learning rates can help the model converge faster, while in the later stages of training, the lower learning rate can help the model adjust to parameters more easily in order to achieve better performance. Finally, the trained model is used for experimental verification to demonstrate the accuracy of the proposed method in this paper.

## 4. Experimental Procedure and Results

**Figure 11:**
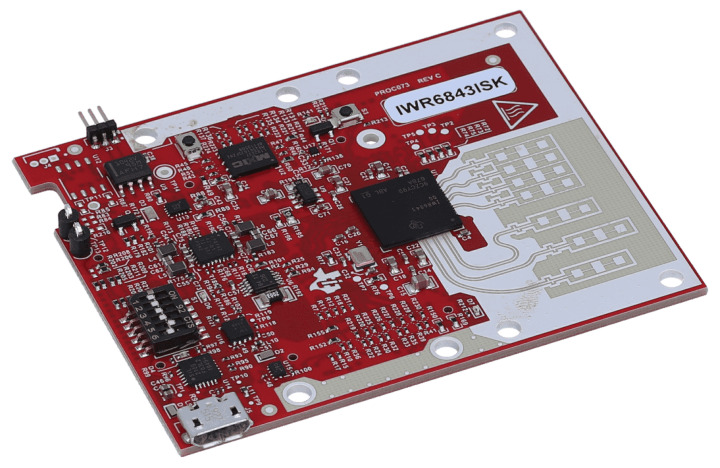
IWR6843ISK radar.

In the experiment, we use the Texas Instruments millimeter-wave FMCW IC (IWR6843ISK), which works in the 60 GHz frequency band. Figure 11 shows the IWR6843ISK radar used in this experiment. In the experiment, IWR6843ISK is used to capture ADC data and transfer to PC through the UART interface.

In order for millimeter-wave radar to better detect human body features inside the vehicle, we make selections for the Chirp configuration, to accurately detect the micro motion of vehicle occupants. The parameter settings for Chirp are shown in Table 2, and the parameter settings for data sample are shown in Table 3.

The range resolution of FMCW millimeter-wave radar can be represented as the following:

(8) $$R_{res}=\frac{c}{2B}$$

By increasing the signal bandwidth B of the radar, the range resolution $R_{res}\;$ of the radar can be directly improved. The radar range resolution in this paper is 3.82 cm.

**Figure 12:**
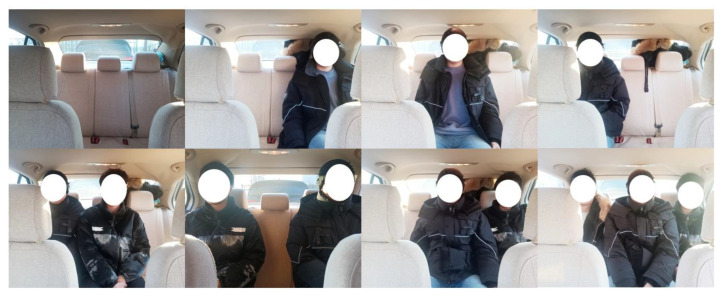
Experimental scenario.

The test system includes a radar module and a PC module, two modules connected by the USB. Figure 12 shows the experimental scenario and different seating situations.

**Figure 13:**
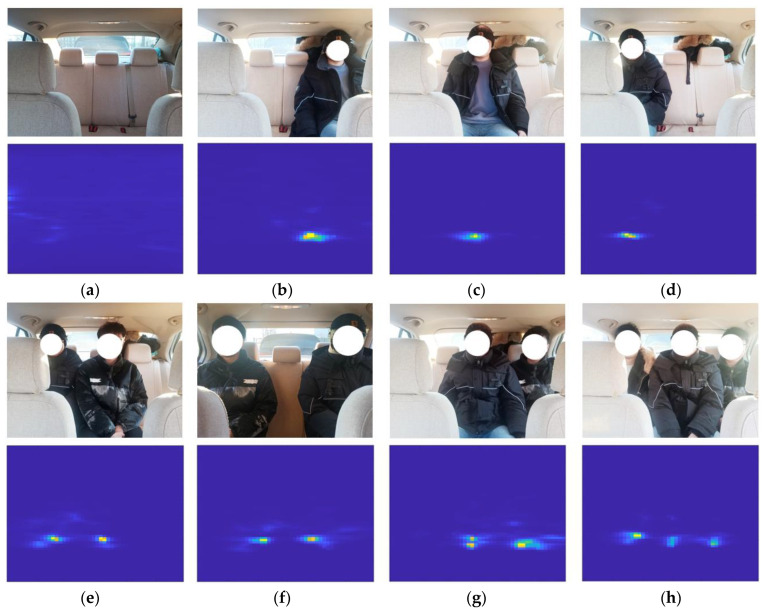
Heatmaps under different seating conditions: (**a**) no one in the seat, (**b**) seating case is 001, (**c**) seating case is 010, (**d**) seating case is 100, (**e**) seating case is 110, (**f**) seating case is 101, (**g**) seating case is 011, (**h**) seating case is 111.

For different seating situations, the radar module is used to collect range–azimuth heatmaps. Figure 13 shows the corresponding radar heat map images under different seating conditions.

**Figure 14:**
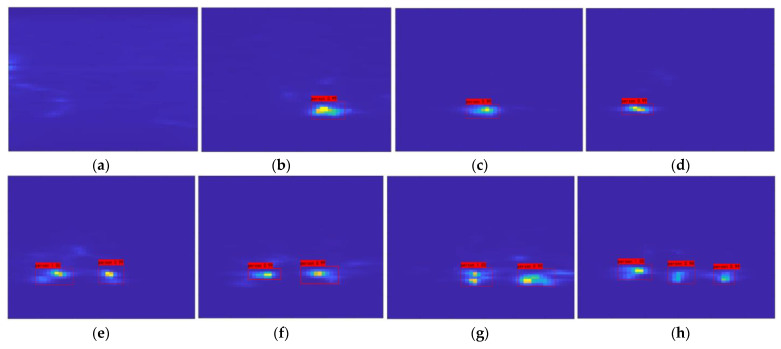
Faster R-CNN training results: (**a**) case 000 result, (**b**) case 001 result, (**c**) case 010 result, (**d**) case 100 result, (**e**) case 110 result, (**f**) case 101 result, (**g**) case 011 result, and (**h**) case 111 result.

The millimeter-wave radar heatmap is collected and organized under different seating conditions, and the dataset is placed into Faster R-CNN neural networks for learning. After learning is complete, the validation dataset is placed into the trained neural network for validation. Figure 14 shows the validation results for eight different seating cases, and the results show that the model can obtain a high recognition accuracy.

In order to objectively and fairly evaluate the in-vehicle occupant detection system based on Faster R-CNN designed in this article, we compared the proposed method with traditional methods using the same sensor. Here, SVM was chosen as a representative model of traditional machine learning.

SVM is a binary classification machine learning model, mainly used for data classification problems in the field of object recognition. It belongs to a supervised learning algorithm, and its basic idea is to find an optimal hyperplane that maximizes the separation of samples from different classes. SVM has shown good results and is widely applied in signal processing, image classification, and other fields.

In the experiment, we set the epoch to 300, the input image resolution to 894*560, and the number of channels to three. The test dataset was divided into three parts: training, validation, and testing. Among them, (training + validation)–testing = 9:1 and training–validation = 9:1. For different seating situations, 40 real-time heat maps were collected for each seating situation, with a total of 320 images used as the validation set to verify the accuracy of different algorithms.

**Figure 15:**
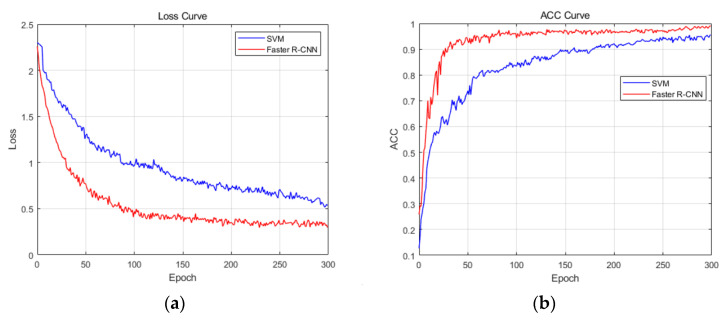
(**a**) Loss curve at different models; (**b**) ACC curve at different models.

Figure 15 shows the loss curve and the ACC curve under two different models.

From the loss curve, as the epoch increases, the loss function of the two models initially converges faster and then becomes smoother. In comparison, the convergence speed of Faster R-CNN is faster and the convergence is better. For the ACC curve, both models have improved accuracy with the increase in epochs. The initial stage has a faster increase rate and gradually becomes smoother, but there is still an upward trend. Table 4 shows the validation set accuracy of two models at different epochs. In comparison, Faster R-CNN has higher accuracy and better model performance.

## 5. Conclusions

In this paper, we propose a method that can be used for vehicle occupant detection using millimeter-wave radar. Firstly, we collect raw radar signals and process them through a radar signal processing algorithm to obtain a range–azimuth heatmap of the target inside the vehicle, visually displaying the current presence status of occupants. Then, based on the range–azimuth heatmap, we extract feature information from a heatmap-based Faster R-CNN network to predict the number and location of occupants. Through experiments, we detect different seating situations in the rear seats and validate the feasibility, accuracy, and effectiveness of the system for the task of vehicle occupant detection.

As this system is not integrated into an embedded system, it still needs to be manually operated and displayed on the computer side. In the future, we can integrate the signal processing part and the seating situations detection part into the embedded system, thereby achieving true miniaturization and portability of the vehicle occupant detection system based on millimeter-wave radar.

## Figures and Tables

**Table 1:** Possible seating options for the three rear seats.

Seating Situation	Occupied Seat Number		
	1	2	3
000	×	×	×
001	×	×	√
010	×	√	×
100	√	×	×
011	×	√	√
101	√	×	√
110	√	√	×
111	√	√	√

**Table 2:** Chirp parameter settings.

Parameter	Numerical Value
Start frequency (GHz)	60
Chirp rate (MHz/μs)	98
ADC start-up time (μs)	10
Ramp end time (μs)	40
Chirp cycle (μs)	340

**Table 3:** Data sample setting.

Parameter	Numerical Value
Sampling numbers	64
Frequency of Sample(ksps)	2200
Chirp numbers/frame	512
Frame cycle(ms)	160

**Table 4:** Validation set accuracy at different models.

	Epoch	50	100	150	200	250	300
Model							
SVM		73.92%	83.51%	89.30%	92.15%	94.01%	95.52%
Faster R-CNN		92.51%	96.14%	97.05%	97.98%	98.54%	99.04%

## Data Availability

The datasets in this study will not be made public for personal reasons.
